# Prolonged antibiotic therapy increased necrotizing enterocolitis in very low birth weight infants without culture-proven sepsis

**DOI:** 10.3389/fped.2022.949830

**Published:** 2022-09-06

**Authors:** Keran Zhu, Hui Gao, Liping Yuan, Lili Wang, Fang Deng

**Affiliations:** ^1^The First Affiliated Hospital of Anhui Medical University, Hefei, China; ^2^Anhui Provincial Children’s Hospital, Hefei, China

**Keywords:** necrotizing enterocolitis, very low birth weight (VLBW), initial empirical antibiotic therapy, antibiotic use duration, hospital stays

## Abstract

**Objectives:**

We aimed to identify the factors associated with necrotizing enterocolitis (NEC) and to assess the associations of the initial empirical antibiotic therapy (IEAT) duration and antibiotic therapy duration/hospital stay ratio (A/H ratio) before NEC with subsequent NEC in very low birth weight (VLBW) infants with gestational age less than 32 weeks without proven sepsis.

**Methods:**

A retrospective study was conducted at the NICU of the First Affiliated Hospital of Medical University of Anhui province from June 2015 to May 2022, and 567 VLBW infants with gestational age less than 32 weeks were included in the study. We divided the VLBW infants into those with and without NEC according to modified Bell’s criteria. We then used descriptive statistics to identify the factors associated with NEC and multivariate analyses to evaluate the associations of IEAT duration and A/H ratio with the occurrence of NEC.

**Results:**

Of the 567 VLBW neonates admitted to our center, 547 survived and reached the normal discharge criteria. Fifty-one infants (8.99%) were diagnosed as showing NEC. Infants with NEC had a longer total parenteral nutrition time, total enteral nutrition time, and IEAT duration, as well as a higher A/H ratio than those without NEC. In multivariate analyses adjusted for the other factors, IEAT duration was associated with an increased odds of NEC [odds ratio (OR) = 1.267; 95% confidence interval (CI), 1.128–1.423], and the A/H ratio was also associated with increased odds of NEC (OR = 8.718; 95% CI, 2.450–31.030). For the A/H ratio, the area under the curve (AUC) was 0.767 and the ideal cutoff was 0.357, and the sensitivity and specificity were 0.843 and 0.645, respectively.

**Conclusion:**

Prolonged antibiotic therapy may increase the risk of NEC in VLBW infants with a gestational age of fewer than 32 weeks and should be used with caution.

## Introduction

Necrotizing enterocolitis (NEC) is a severe disease that mainly occurs in premature neonates, especially in very low birth weight (VLBW) infants. This inflammatory disease occurs in the neonatal period and is characterized by abdominal distension, vomiting, and bloody stools with necrotizing changes, especially in premature infants. The clinical symptoms and signs are variable and include feeding intolerance, abdominal distension, bloody stools, shock, and sepsis. The treatment of NEC can involve conservative and surgical methods according to Bell’s criteria, and patients may show residual complications, such as bowel restriction, short bowel syndrome, and cholestasis. In addition to extremely high morbidity and mortality and high costs, long-term complications include strictures and adhesions of the intestine, cholestasis, short bowel syndrome, failure to thrive, and neurodevelopmental delay (1).

The pathogenesis of NEC remains a topic of debate, and the relationships among the contributing factors are complex and unclear. Gestational age (GA), birth weight (BW), and feeding style are considered certain risk factors for NEC. However, few studies have reported the antenatal and maternal factors influencing the occurrence of NEC. In one previous study, subclinical chorioamnionitis was shown to be related to a higher incidence of morbidity in the preterm gut. Antibiotic use is another point that deserves attention and is a controversial topic. Deficient antibiotic use can result in inadequate elimination of pathogens while excessively prolonged antibiotic use could change the initial gut flora, and altered gut colonization can play a direct role in the pathogenesis of NEC. Prolonged empirical antibiotic exposure after birth in VLBW infants was associated with increased odds of the composite outcome, including necrotizing enterocolitis, which was worth our attention (2).

Preterm infants have their initial microecological diversity, and postnatal antibiotic use can change the gut environment. Cotton et al. found that prolonged initial antibiotic use was related to increasing NEC and death (3). Kuppala et al. reported that the outcome of prolonged initial empirical antibiotic use was a combination of NEC and death (4). Raba et al. performed a case-control retrospective study to demonstrate that prolonged exposure to antibiotics was associated with an increased risk of NEC among VLBW infants (5). Greenberg et al. studied extremely premature infants (GA, 22–28 weeks) and found that prolonged antibiotic use was not associated with NEC and death [adjusted odds ratio (OR), 1.17; 95% confidence interval (CI), 0.99–1.40; *p* = 0.07] (6). Flannary et al. conducted a retrospective cohort study and found a small but significant decrease in the rate of prolonged antibiotic use in VLBW infants (*p* = 0.02), but not in extremely low birth weight (EVLBW) infants (*p* = 0.22) (7). In this study, we aimed to identify the factors associated with necrotizing enterocolitis (NEC) and to assess the associations of the initial empirical antibiotic therapy (IEAT) duration and antibiotic therapy duration/hospital stay ratio (A/H ratio) before NEC, with subsequent NEC in very VLBW infants with gestational age less than 32 weeks without proven sepsis.

## Materials and methods

### Study population

From June 2015 to May 2022, 567 VLBW infants with gestational age less of than 32 weeks without culture-proven sepsis were admitted to the NICU of the First Affiliated Hospital of Medical University of Anhui Province. We used the penicillin antibiotics and third-generation cephalosporin as the initial empirical antibiotic therapy (IEAT) and subsequent antibiotic therapy. Approval for this study was acquired from the Medical ethics committee of the First Affiliated Hospital of Anhui Medical University. After excluding infants who died; those with hematologic disease, abdominal deformities, chest deformities, or other congenital anomalies; and infants whose parents desisted. A total of 51 infants (8.99%) were diagnosed as NEC (≥ Bell IIA stage) according to modified Bell’s criteria, which was based on the presence of one or more clinical symptoms (e.g., abdominal distension, tenderness, gastric vomiting, and bloody stools) and radiographic findings (e.g., dilatation of the intestine, hepatobiliary gas, portal vein gas, and pneumoperitoneum).

### Information extraction

All information was abstracted from the medical records. NEC was defined according to modified Bell’s criteria. At delivery, data regarding the infant’s sex (male vs. female), GA, BW, fetal growth restriction (FGR), 1- and 5-min Apgar scores, cesarean section (C-section), premature rupture of membranes (PROM), chorioamnionitis, and amniotic fluid pollution were obtained. Patent ductus arteriosus (PDA) was defined based on the clinical symptoms and the findings of vascular ultrasound examinations.

We defined prolonged initial antibiotic treatment as ≥ 5 days of initial antibiotic treatment with sterile culture results. The time of initial antibiotic treatment was defined as the number of days, after which the initially administered antibiotics were discontinued. The time of antibiotic exposure was defined as the total number of days, for which the infant was administered antibiotics before NEC. We also aimed to determine the initial empirical antibiotic usage and antibiotic exposure before NEC.

Early-onset sepsis (EOS) was diagnosed during the first 3 days after birth (< 3 days) based on the positive blood culture results within 3 postnatal days and treatment for ≥ 5 days or clinical symptoms with a negative culture result; late-onset sepsis (LOS) was defined as a positive blood, cerebrospinal fluid (CSF), or urine culture or clinical symptoms with a negative culture result after 3 postnatal days.

The definition of sepsis was SIRS in the presence of or as a result of suspected or proven infection (8). Neonatal sepsis was defined by the presence of at least two clinical symptoms and at least two laboratory signs in the presence of or as a result of suspected or proven infection [positive culture, microscopy, or polymerase chain reaction (PCR)]. Definition of proven sepsis was that clinical and laboratory findings were present, and demonstration of the pathogenic microorganism in cultures taken from the sterile field. Suspected sepsis and clinical sepsis were diagnosed based on the clinical symptoms and laboratory examinations.

Total enteral nutrition (TEN) was defined as a total intake of breast milk or formula milk of at least 120 ml/kg/d, and the time of TEN referred to the number of days, on which the neonate received at least 120 ml/kg/d of breast milk or formula milk.

The definition of total parenteral nutrition (TPN) in our study was defined as the time of total parenteral nutrition before NEC.

### Statistical analysis

Mean and SD were described for continuous variables if they showed a normal distribution; Student’s *t*-test was used to compare these continuous variables across different patient groups. The median and interquartile ranges were used to present continuous variables that showed a skewed distribution; the Mann–Whitney test was used to compare these skewed variables across different groups. Categorical variables were summarized as frequencies and percentages. The frequencies of categorical variables were compared using the chi-square and Fisher’s exact test as appropriate. A binary logistic model that included variables significantly associated with NEC was used to estimate adjusted ORs and 95% CIs for NEC. All statistical analyses were performed with SPSS 16.0 and Stata 13.0, and *p* < 0.05 obtained with two-tailed tests was considered statistically significant.

## Results

### Demographic and clinical characteristics

Table 1 shows the maternal characteristics of the 567 VLBW infants. The average maternal age was 30.40 ± 4.92 years; 468 mothers (82.54%) had singleton pregnancies, while 293 (51.68%) neonates were delivered by cesarean delivery. A total of 352 (68.78%) mothers received perinatal corticosteroids for promoting fetal lung maturation. Maternal hypertension was present in 19.75% of the cases, while PROM (≥ 24 h) complicated 21.69% of the pregnancies. Chorioamnionitis and amniotic fluid pollution were reported in 4.76% and 2.29% of the pregnancies, respectively.

**TABLE 1:** Clinical characteristics of VLBW infants with and without NEC.

Characteristics	All VLBW infants (*N* = 567)	VLBW infants without NEC (*n* = 516)	VLBW infants with NEC (*n* = 51)	Statistical parameter	Statistical parameter (*P-value*)
Maternal age (years)	30.40 ± 4.92	30.28 ± 4.87	31.54 ± 5.38	−1.661	0.097
C-section (%)	293 (51.68)	270 (52.33)	23 (45.10)	0.971	0.379
Singleton (%)	468(82.54)	426(82.56)	42(82.35)	0.103	0.950
Antenatal steroid use (%)	390 (68.78)	352 (68.22)	38 (74.51)	0.922	0.631
Hypertension (%)	112 (19.75)	102 (19.77)	10 (19.61)	0.001	0.978
PROM (≥ 24 h) (%)	123 (21.69)	109 (21.12)	14 (27.45)	1.094	2.096
Chorioamnionitis (%)	27 (4.76)	24 (4.65)	3 (5.88)	0.155	0.726
Amniotic fluid pollution (%)	13(2.29)	12(2.33)	1(1.96)	0.028	1.000
Gestational age (weeks)	29.71 ± 1.34	29.70 ± 1.39	29.73 ± 1.26	−0.122	0.903
Birth weight (grams)	1249.05 ± 159.79	1247.22 ± 161.39	1267.45 ± 142.73	−0.862	0.389
1-min Apgar score (scores)	6.58 ± 2.15	6.55 ± 2.15	6.84 ± 2.20	−0.929	0.353
5-min Apgar score (scores)	8.30 ± 1.55	8.28 ± 1.53	8.45 ± 1.82	−0.733	0.464
SGA (%)	21 (3.70)	18 (3.49)	3 (5.88)	0.764	0.424
NEC appearance time (days)	–	–	36.59 ± 18.70	–	–
Initial feeding days (days)	2.13 ± 0.96	2.11 ± 0.95	2.31 ± 0.99	−1.440	0.150
TPN time (days)	3.79 (2.00,5.00)	3.47 (2.00,5.00)	6.98 (3.00,8.50)	−5.509	0.000
TEN time (days)	12.65 (9.00,15.00)	12.42 (9.00,15.00)	15.15 (9.00,18.00)	−2.467	0.014
IEAT duration (days)	4.83 (3.00,7.00)	4.40 (3.00,6.00)	6.73 (5.00,8.00)	−6.019	0.000
Antibiotic use duration before NEC occurrence (days)	16.98 (7.00,21.00)	16.98 (7.00,22.00)	17.00 (11.00,20.00)	−2.019	0.052
Hospital stays before NEC (days)	44.24 (34.00,53.50)	44.99 (35.00,54.00)	36.59 (23.50,50.00)	2.366	0.000
Total hospital stays (days)	45.70 (36.00,55.00)	45.27 (35.00,54.75)	50.00 (40.00,60.00)	−2.199	0.028
A/H ratio before NEC	0.41 (0.19,0.53)	0.39 (0.18,0.50)	0.57 (0.37,0.65)	−4.490	0.000

Data are presented as mean ± standard deviation or median (interquartile range). A/H ratio, antibiotic use time/hospital stay ratio; FGR, fetal growth restriction; IEAT, initial empirical antibiotic therapy; PROM, premature rupture of membranes; SGA, small for gestational age; TEN, total enteral nutrition; TPN, total parenteral nutrition.

Table 2 shows the neonatal characteristics of the 567 VLBW infants. Fifty-one infants (8.99%) were diagnosed as showing NEC. The GA of the study cohort was 29.71 ± 1.34 weeks, and their BW was 1,249.05 ± 159.79 g (range, 1,000–1,495 g); the 1- and 5-min Apgar scores were 6.58 ± 2.15 and 8.30 ± 1.55, respectively. Twenty-one neonates (3.70%) were small for GA. The initial feeding days in the NEC group were longer than that in the no-NEC group (median: 2.11 days vs. 2.31 days). The time of TPN in the NEC group was longer than that in the no-NEC group (median: 6.98 days vs. 3.47 days), and the time of TEN in the NEC group was also longer than that in the no-NEC group (median: 15.15 days vs. 12.42 days). IEAT duration in the NEC group was longer than that in the no-NEC group (median: 6.73 days vs. 4.40 days). The A/H ratio before NEC occurrence in the NEC group was larger than that in the no-NEC group (median: 0.57 vs. 0.39).

**TABLE 2:** Comparison of prolonged IEAT and A/H ratios before NEC according to the outcomes of NEC and death.

Characteristics	No outcome	With outcome	Z/χ ^2^	*P*
NEC or death, N	512	55		
Prolonged IEAT, n (%)	175 (34.18)	34 (61.82)	16.301	0.001
IEAT duration (days)	4.52 (3.00,7.00)	5.48 (3.00,7.00)	−2.156	0.031
A/H ratio before NEC	0.38 (0.18,0.50)	0.65 (0.40,0.84)	−5.477	0.000
NEC, N	516	51		
Prolonged IEAT, n (%)	170(33.20)	39 (70.91)	37.779	0.000
IEAT duration (days)	4.40 (3.00,6.00)	6.73 (5.00,8.00)	−6.050	0.000
A/H ratio before NEC	0.39 (0.18,0.50)	0.57(0.37,0.65)	−4.471	0.000
Death, N	547	20		
Prolonged IEAT, n (%)	204 (37.29)	5 (25.00)	1.253	0.263
IEAT duration (days)	4.71 (3.00,7.00)	3.00 (1.00,4.50)	−0.744	0.439
A/H ratio before NEC	0.40 (0.19,0.50)	0.57 (0.37,0.65)	−6.279	0.000

Data are presented as mean ± standard deviation or median (interquartile range). A/H ratio, antibiotic use time/hospital stay ratio; IEAT, initial empirical antibiotic therapy; NEC, necrotizing enterocolitis.

### Association between antibiotic use and the risk of necrotizing enterocolitis

The logistic regression model showed that IEAT duration increased the risk of NEC by 1.295-fold. The risk persisted after adjusting for TPN time, TEN time, and the A/H ratio before NEC (OR = 1.267; 95% CI, 1.128–1.423). Meanwhile, the logistic regression model showed that the A/H ratio before NEC increased the risk of NEC by 12.654-fold. The risk persisted after adjusting for TPN time, TEN time, and IEAT duration (OR = 8.718; 95% CI, 2.450–31.030) (Tables 3, 4).

**TABLE 3:** Association between IEAT and NEC analyzed by multiple logistic regression.

IEAT duration	OR	95% CI	*P-value*
Crude NEC	1.295	1.172–1.431	0.000
Adjusted NEC*	1.267	1.128–1.423	0.000

CI, confidence interval; IEAT, initial empirical antibiotic therapy; NEC, necrotizing enterocolitis; OR, Odds ratio. *Adjusted for parenteral nutrition time, total enteral nutrition time, and antibiotic therapy/hospital stays ratio before NEC.

**TABLE 4:** Association between A/H ratio and NEC analyzed by multiple logistic regression.

A/H ratio before NEC	OR	95% CI	*P-value*
Crude NEC	12.654	4.725–33.840	0.000
Adjusted NEC^#^	8.718	2.450–31.030	0.001

CI, confidence interval; NEC, necrotizing enterocolitis; OR, odds ratio. ^#^Adjusted for total parenteral nutrition time, total enteral nutrition time, and initial empirical antibiotic therapy duration.

### Predictive value of the A/H ratio

**FIGURE 1:**
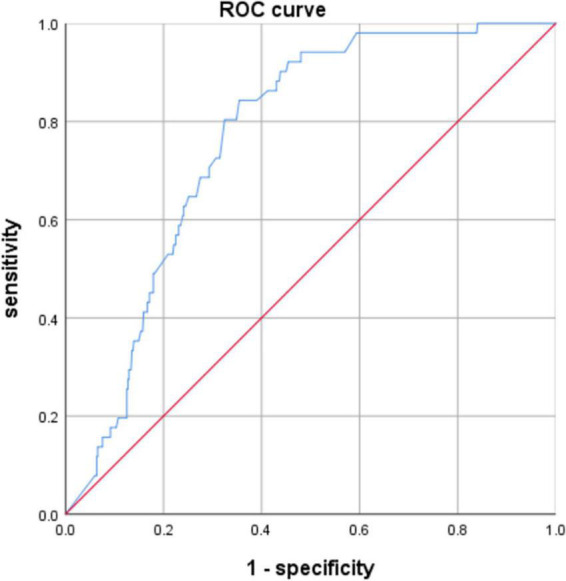
ROC curve of the A/H ratio.

The AUC of the A/H ratio was 0.767 with a cutoff value of 0.357. At a Youden’s index of 0.488, the sensitivity and specificity were 0.843 and 0.645, respectively (*P* = 0.000; 95% CI, 0.715–0.818) (Table 5 and Figure 1).

**TABLE 5:** Receiver operating characteristic (ROC) analysis values of A/H ratios.

Comparison	AUC	Standard error	*P-value*	95% CI	Sensitivity	Specificity	Youden’s index
A/H ratio	0.767	0.026	0.000	0.715–0.818	0.843	0.645	0.488

A/H ratio, antibiotic use time/hospital stay ratio; AUC, area under the curve; OR, odds ratio.

## Discussion

Our study indicated that prolonged duration of antibiotics was associated with NEC. Our data suggested that IEAT duration and the A/H ratio might be the risk factors of NEC in VLBW infants with gestational age of less than 32 weeks without proven sepsis. IEAT duration increased the risk of NEC by 1.267-fold and A/H ratio before NEC increased the risk of NEC by 8.718-fold. A ratio of more than 0.357 before NEC might increase the risk of NEC.

Our results were similar to the findings of several recent investigations about antibiotics and NEC. Esmaeilizand et al. conducted a retrospective case-control study to examine the relationship between antibiotic exposure and stages II and III NEC, and they concluded that antibiotic exposure for more than 5 days in preterm infants with GA of less than 29 weeks was associated with the incidence of NEC (9). In a quantitative cross-sectional study conducted over 5 years in a NICU and including 266 cases, Torres et al. concluded that IEAT and antimicrobial therapy longer than 5 days were associated with sepsis and NEC, but not with mortality (10). Martinaz et al. included 901 infants, of which 67 infants received IEAT, and found a 50% increase in nutrition usage duration and a fourfold greater prevalence in the early initial empirical antibiotic exposure group (11). Rina et al. included thirteen studies, including 7,901 participants, and that the IEAT (≥ 5 days) was associated with an increased risk of NEC (12). In our study, the average IEAT time was 4.40 days in the no-NEC group and 6.73 days in the NEC group. Our results were similar to the findings of a previous study, which indicated that prolonged IEAT was the risk factor of the disease that deserved attention.

For the relationship between the total duration of antibiotic use and NEC, different studies have reported contradictory findings. To determine whether antibiotic use in the first 14 postnatal days is associated with adverse outcomes, Cantey et al. evaluated 374 VLBW infants and found that antibiotic usage in this period was associated with LOS, NEC, and death after controlling for the severity of illness (13). However, a cohort study by Greenberg et al. included 5,750 extremely low birth weight (EVLBW) infants (22–28 weeks) without a major defect born from 2008 to 2014 at 13 centers and concluded that prolonged early antibiotics were not significantly associated with increased odds of death or NEC (6). Raba et al. also found that the cumulative total number of days of antibiotic exposure was associated with an increased risk of developing NEC (5). Chen et al. included 132 VLBW infants and found each additional day of antibiotic treatment was associated with increased odds of NEC (OR, 1.278; 95% CI, 1.025–1.593) (14). The findings of our study agreed with the results of these studies which suggested that the A/H ratio before NEC could increase the risk of NEC.

Antibiotic administration was known to perturb the composition of the intestinal microbiota, resulting in suppression of beneficial bacteria and increased numbers of potentially pathogenic bacteria such as *Klebsiella, Enterobacter, Citrobacter*, and *Pseudomonas*. The choice of antibiotic duration was likely to affect the colonization of the neonatal intestine. Prolonged antibiotic therapy could shift the balance of intestinal microbiota toward pathogenic factors, leading to NEC. The main reason for initial empirical antibiotic prophylaxis was to address the possibility of maternal infection-induced preterm labor, such as PROM, chorioamnionitis, amniotic fluid pollution, and maternal fever. Thus, IEAT could be defined as treatment based solely on a clinical suspicion of infection without a positive culture result. We found that the duration of IEAT in the NEC group in the present study was 6.73 (5.00, 8.00) days, while the corresponding value in the no-NEC group was 4.40 (3.00, 6.00) days, and that the IEAT duration was an independent risk factor for NEC (adjusted OR = 1.267; 95% CI, 1.128–1.423; *P* = 0.000), so we speculated that prolonged IEAT might shift the composition of the intestinal microbiota, resulting in increased pathogenetic bacteria.

Prolonged antibiotic administration before NEC was also used as an anti-infective treatment. However, the prolonged use of antibiotics could impair the homeostasis of intestinal flora. Antibiotic therapy was known to alter the colonization of the gastrointestinal tract and predispose individuals to the emergence of pathogens and resistant organisms. Animal studies of intestinal development have underscored the abnormal interactions in the intestinal epithelium and the interruption of luminal bacterial colonization in animals housed in germ-free conditions or treated with prolonged antibiotics. Activation of toll-like receptors by commensal bacteria appears to be critical for affording protection against gut injury and associated mortality (15, 16). The TLR4-mediated imbalance between proinflammatory and anti-inflammatory signaling in the premature intestinal epithelium led to the development of NEC (17). Microbial dysbiosis preceding NEC in preterm infants was characterized by increased relative abundances of *Proteobacteria* and decreased relative abundances of *Firmicutes* and *Bacteroidetes* (18). Microbiome optimization may provide a novel strategy for preventing NEC. The lack of bacterial species diversity and the abundance of *Proteobacteria* species associated with the widespread use of antibiotics may predispose neonates to inflammatory stimulation, which may help explain the susceptibility of premature newborns to NEC (19). Prolonged antibiotic exposure may reduce the biodiversity of the intestinal microbiota and may predispose preterm infants to NEC (20). In neonatal mice, systemic antibiotic trent impaired the intestinal development by reducing intestinal cell proliferation, villi height, crypt depth, and goblet and Paneth cell numbers; the pups who received antibiotics resulted in NEC-like intestinal injury in more than half the pups, likely due to a reduction in mucous-producing cells affecting microbial-epithelial interactions (21). These data support a novel mechanism that could explain why preterm infants exposed to prolonged antibiotics after birth had a higher incidence of NEC and other gastrointestinal disorders. Prolonged antibiotic therapy before NEC also affected the gut flora, since the A/H ratio was another independent risk factor for NEC in our study (OR = 8.718; 95% CI, 2.450–31.030; *P* = 0.001). When the A/H ratio was more than 0.357 before NEC occurrence, the risk of NEC increased, highlighting the importance of reasonable application of antibiotics during hospitalization and the potential ability of prolonged antibiotic use during the hospital stay to increase the risk of NEC.

This study had several limitations that require consideration. First, the study was based on data obtained from a single center, and the inclusion of data from more centers might reveal more perinatal risk factors for NEC. Second, the number of cases was relatively small, and a larger number of patient cases was required to study the differences between the two groups. Third, some patients were lost to follow-up. Thus, a prospective cohort study with good quality control would be warranted. Prolonged antibiotic treatment was primarily administered to account for the possibility of maternal infection-induced preterm labor. The initial empirical or antibiotic prophylaxis was defined properly as treatment based on clinical suspicion of infection without a positive culture result, and subsequent prolonged antibiotic was used based on clinical experience which was lack of laboratory evidence.

Our findings indicated that prolonged antibiotic usage was associated with the high risk of NEC in VLBW infants, prolonged antibiotic therapy might increase the risk of NEC in VLBW infants with gestational age of less than 32 weeks and should be used with caution. Thus, in neonatal clinical practice, antenatal and postnatal diseases should be evaluated carefully and the duration of antibiotic use should be strictly controlled. Rational use of antibiotics might reduce the risk of NEC in VLBW infants.

## Data availability statement

The raw data supporting the conclusions of this article will be made available by the authors, without undue reservation.

## Author contributions

KZ: study concept and design and drafting of the manuscript. LY: acquisition of data. HG: analysis and interpretation of data and statistical analysis. LW: critical revision of the manuscript for important intellectual content. FD: administrative, technical and material support, and study supervision. All authors contributed to the article and approved the submitted version.
